# Association between angiotensin-converting enzyme inhibitor-induced cough and the risk of lung cancer: a Mendelian randomization study

**DOI:** 10.3389/fphar.2023.1267924

**Published:** 2023-09-20

**Authors:** Taikang Yao, Zhenchao Wu, Zilu Wang, Liting Chen, Beibei Liu, Ming Lu, Ning Shen

**Affiliations:** ^1^ Department of Pulmonary and Critical Care Medicine, Peking University Third Hospital, Beijing, China; ^2^ Peking University Health Science Center, Peking University, Beijing, China

**Keywords:** angiotensin-converting enzyme inhibitor, angiotensin-converting enzyme inhibitor-induced cough, lung cancer, lung adenocarcinoma, Mendelian randomization

## Abstract

**Background:** Observational studies and meta-analyses have demonstrated a positive correlation between the use of angiotensin-converting enzyme inhibitors (ACEIs) and lung cancer. However, the findings remain controversial; furthermore, the relationship between ACEI-induced cough and lung cancer development remains unknown. We used Mendelian randomization (MR) to verify the association between ACEI use, ACEI-induced cough, and the risk of lung cancer.

**Methods:** We performed a two-sample MR analysis to determine the unconfounded relationships between ACE inhibition, which mimics the effects of ACEIs, and genetic proxies for ACEI-induced cough and lung cancer. Single nucleotide polymorphisms that imitate ACE receptors and ACEI-induced cough were collected and integrated into a meta-analysis of existing genome-wide association studies for various lung cancers. The relationship was quantified using inverse variance weighting, weighted median, and MR-Egger methods.

**Results:** A statistically significant association was observed between ACE inhibition and the risk of small cell lung cancer for Europeans (excluding rs118121655/rs80311894). Associations were identified between ACEI-induced cough and the risk of lung cancer for Europeans, although not for Asians, and between ACEI-induced cough and lung adenocarcinoma (excluding rs360206).

**Conclusion:** Our findings reveal a relationship between ACE inhibition and lung cancer development, as well as a significant association between ACEI-induced cough and a higher risk of lung cancer for Europeans. Patients with hypertension who experience dry cough as a side effect of ACEI use should consider switching to an alternative antihypertensive treatment.

## 1 Introduction

The incidence of hypertension and heart failure is increasing worldwide. Nearly 1.28 billion patients have hypertension (as of 2019), (NCD Risk Factor Collaboration NCD-RisC, 2021), and 64.3 million patients have heart failure (as of 2017) (Bragazzi et al., 2021) worldwide. These diseases contribute to heavy healthcare and economic burdens. Angiotensin-converting enzyme (ACE) inhibitors (ACEIs) are first-line cornerstone drugs that are widely used to treat hypertension and heart failure. ACEIs are markedly effective for the treatment of hypertension (George et al., 2010). However, the side effects of ACEIs remain controversial. Many clinical studies have shown that ACEIs can lead to the development of dry cough in up to 20% of patients (Israili and Hall, 1992; Unger et al., 2020). The development of cancer as a potential adverse event caused by ACEI use has received increasing attention from clinicians and patients. Recent clinical studies have shown that lung cancer is a notable adverse event attributable to ACEI use (Hicks et al., 2018; Lin et al., 2020; Kristensen et al., 2021). A recent meta-analysis (Wu et al., 2023) by the authors of the present study (Wu and Yao) demonstrated that ACEI use is a greater risk factor for lung cancer (up to 1.6%) than angiotensin receptor blocker use, especially among Asian patients.

Although the results of many clinical studies support this phenomenon, no randomized controlled trials (RCTs) have been conducted to confirm the causal association between ACEI use and lung cancer risk. Previously conducted RCTs of ACEIs only evaluated the effects of ACEIs on cardiovascular and renal endpoints (The GISEN Group, 1997; Fox et al., 2003); the incidence rate of cancer was not included in those studies. Various factors might cause difficulty implementing RCTs. During previous observational studies, the interference of potential confounding factors may have been the main reason for inconsistent results. In addition, verifying the causal relationship between risk factors and outcomes is difficult; furthermore, reverse causality may also indicate this relationship. Under these conditions, Mendelian randomization (MR), as a natural randomized controlled study, provides a new approach that utilizes drug target-related genetic variants to simulate drug effects or the risk of adverse events, thereby solving the aforementioned problems to some extent (Gill et al., 2019). According to the Mendelian genetic law, parental alleles are randomly assigned to offspring. Genetic variants are unlikely to be affected by the post-birth environment, socio-economic status, and behavioral factors. Furthermore, with MR, the causal time sequence is reasonable and the study design minimizes residual confounding factors. Therefore, the study of disease associations using genes as instrumental variables has recently become a hot topic among epidemiological researchers (Emdin et al., 2017).

A recent meta-analysis (Wu et al., 2023) revealed that Asians may be at higher risk for lung cancer development with ACEI use. Although some researchers (Hicks et al., 2018; Borghi et al., 2023) found that ACEI-induced cough or carcinogenesis was related to substance P and bradykinin, no relevant clinical studies have supported this finding. Additionally, it is not known whether ACEI-induced cough is associated with lung cancer. Therefore, we aimed to answer the following questions through an MR analysis: do ACEIs cause lung cancer? what is the risk of lung cancer for different races using ACEIs? do ACEIs lead to a certain pathological subtype of lung cancer? is ACEI-induced dry cough related to lung cancer? if ACEI-induced dry cough is related to lung cancer, what are the differences among races or lung cancer types? which body substances are involved in the development of lung cancer caused by ACEI use? and what are the implications regarding medication and management guidance for patients with hypertension using ACEI therapy?

## 2 Materials and methods

### 2.1 Study design

We selected single nucleotide polymorphisms (SNPs) related to ACE inhibition and ACEI-induced cough from previous genome-wide association studies (GWAS) for use as genetic proxies for exposure. The whole-genome detection of ACE inhibition was obtained from a previous study, (Yarmolinsky et al., 2022), and SNPs related to ACEI-induced cough in the European population were obtained from a meta-analysis (Ghouse et al., 2022) of three independent European cohorts. Additionally, SNPs related to ACEI-induced cough in the Asian population were sourced from 396 Chinese individuals (Mu et al., 2020). To estimate the association between exposure and outcomes, we extracted the effect sizes of the selected SNPs from GWAS. Exposure-outcome association was estimated using the effect sizes of the same SNPs from a lung cancer GWAS. All data used in this study are publicly available and were obtained from populations in East Asia and Europe. The present study was conducted in accordance with the observational epidemiology study reporting guidelines set forth in the Strengthening the Reporting of Observational Studies in Epidemiology-Mendelian Randomization reporting guide (Skrivankova et al., 2021).

### 2.2 Instrument selection

Instrumental variables should conform to three key assumptions:1) they associate with the risk factor of exposure (ACE inhibition or ACEI-induced cough); 2) they share no common cause with the outcome (lung cancer); and 3) they do not directly affect the outcome except through the risk factor. To verify the utility of SNPs related to ACE inhibition as targets for ACEIs, we used a two-sample MR approach to estimate their impact on systolic blood pressure (SBP). After determining the impact on SBP, SNPs related to ACE inhibition levels were used to estimate the impact of ACEIs on the development of lung cancer. The strength of each genetic variant was evaluated using F-statistics. Genetic variants with an F-statistic value greater than 10 were included in the analysis. (Pierce et al., 2011). Gene expression in the blood that was not statistically significant (*p* ≥ 0.05) in relation to SBP was excluded from further analyses. During the primary analysis, SNPs that affected SBP were retained. Additionally, we used SNPs related to ACEI-induced cough as a genetic proxy and used a two-sample MR approach to estimate the association between ACEI-induced cough and lung cancer risk. We performed a subgroup analysis to differentiate between ethnicity and lung cancer pathology.

### 2.3 GWAS acquisition

The primary outcome of the MR analysis was lung cancer. To obtain GWAS summary datasets related to lung cancer, we utilized the latest data from the Medical Research Council Integrative Epidemiology Unit (University of Bristol) GWAS database (https://gwas.mrcieu.ac.uk/), which comprises up to 245, 497, 737,356 genetic associations derived from 42,335 GWAS summary datasets (version 7.0.0). Further details regarding each individual GWAS can be accessed using the GWAS database (https://gwas.mrcieu.ac.uk/datasets/). The database was retrieved using the TwoSampleMR package in R studio (R Foundation for Statistical Computing, Vienna, Austria) (Hemani et al., 2018). The details of these studies have been previously described, and the demographic characteristics of the GWAS summary datasets used for the MR analysis appear in Supplementary Table S1. Additionally, for the subgroup MR analysis, the pathological subtypes of lung cancer, including non-small cell lung cancer (NSCLC), adenocarcinoma, lung squamous cell carcinoma, and small cell lung cancer (SCLC), were considered.

### 2.4 Statistical analysis

During the MR analysis, we used an inverse variance-weighted (IVW) approach that considers the multiplicative random effects to meta-analyze the SNP-specific Wald estimates. IVW regression was applied to test the potential causal relationship between ACEI-induced cough (*X*) and lung cancer risk (*Y*), with ACEI-induced cough-related SNPs serving as instrumental variables (Guo et al., 2016). The causal relationship (*β*_*YX*_) between ACEI-induced cough and the risk of lung cancer was estimated as follows using the Wald estimator: *β*_*YX*_ = *β*_*YG*_/*β*_*XG*_, where *β*_*XG*_ is the estimated effect of lung cancer associated with the instrumental variable and *β*_*XG*_ is the estimated effect of ACEI-induced cough on SBP associated with the instrument variable. The MR-Egger regression analysis was performed to estimate the potential pleiotropic effects of SBP-related genetic variants (Bowden et al., 2015).

**FIGURE 1:**
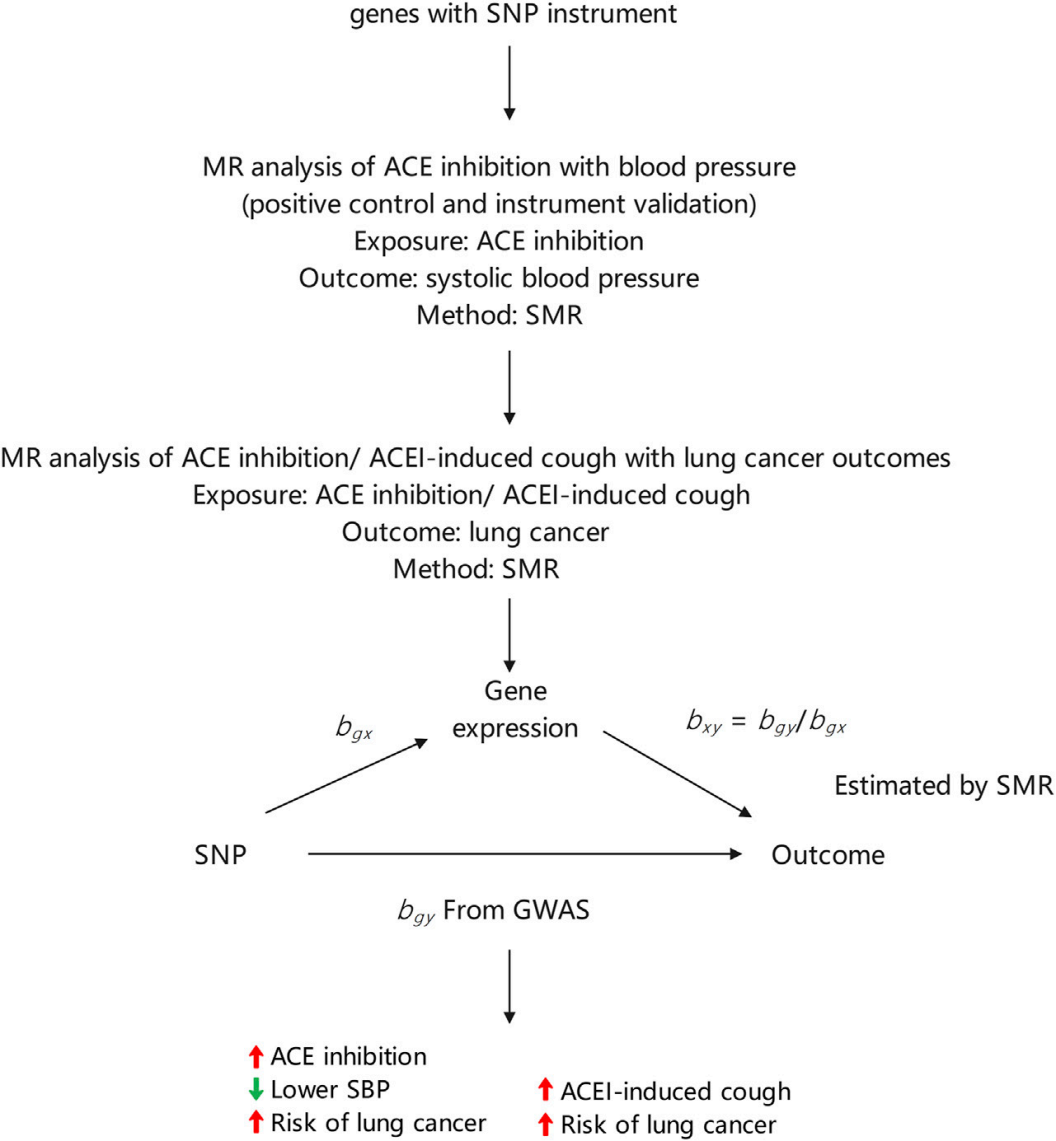
Summary of study design.

The final MR estimate of the relationship between ACEI-induced cough and the risk of lung cancer was based on SNP-specific Wald estimates such as the genetic association with lung cancer divided by the genetic proxy for ACEI-induced cough. Therefore, the effect size estimate in this study implied the direction of the potential association between ACEI-induced cough and the risk of lung cancer. The statistical analysis process is illustrated in Figure 1.

During the sensitivity analysis, different methods were used to control for pleiotropy, including weighted median and Mendelian Randomization Pleiotropy Residual Sum and Outlier methods. A leave-one-out analysis was performed to estimate whether the results were biased or driven by a single SNP. The weighted median estimate was robust despite invalid instruments, providing consistent estimates even when up to 50% of the weight was from invalid SNPs (Bowden et al., 2016). The Mendelian Randomization Pleiotropy Residual Sum and Outlier method can be used to identify outliers with potential pleiotropic levels among multiple genetic variations and provide corrected estimates after removing these outliers (Verbanck et al., 2018). The Cochran Q statistic was calculated to test for heterogeneity (Giri et al., 2020). The pleiotropy of the SNPs was also validated. We summarized hypothetical scenarios that might lead to the observed association between ACE inhibition (or ACEI-induced cough) and the risk of lung cancer (Supplementary Figure S1). All statistical analyses were performed using R studio software and the TwoSampleMR package.

## 3 Results

### 3.1 Selection of genetic instruments

Fourteen SNPs related to ACE inhibition were reported by a previous study (Yarmolinsky et al., 2022). We obtained correlations between SNPs and exposure relevant to the European population from that study. These 14 SNPs were included in the main European population analysis (Supplementary Table S2). The SNPs selected as genetic instruments had F-statistic values greater than 10.

For the MR analysis related to ACEI-induced cough, we selected 11 SNPs from a published GWAS of the Asian population (Chinese) (Mu et al., 2020). From that study, we extracted the corresponding correlations between SNPs and exposure for the Asian population. These 11 SNPs were included in the main Asian population analysis (Supplementary Table S3). Additionally, we selected seven SNPs from a published European GWAS (Ghouse et al., 2022) and extracted correlations between SNPs and exposure relevant to the European population. These seven SNPs were included in the main European population analysis (Supplementary Table S4).

### 3.2 MR analysis of ACE inhibition and lung cancer risk

First, we verified the effect of ACE inhibition on SBP. The MR analysis revealed a negative correlation between ACE inhibition and SBP (trait: ieu-b-38) (odds ratio [OR], 0.768; 95% confidence interval [CI], 0.574–0.961; *p* = 0.007), (Howe et al., 2022), implying that the genetic variation associated with ACE inhibition could be used to mimic the effect of ACEIs. We conducted an in-depth analysis of a European cohort to investigate the potential causal association between ACE inhibition and lung cancer (trait: finn-b-C3_BRONCHUS_LUNG); however, we found no evidence of a causal relationship (OR, 0.959; 95% CI, 0.854–1.063; *p* = 0.429). The results of the leave-one-out analysis were robust (Supplementary Table S5). We also investigated the relationship between ACE inhibition and specific subtypes of lung cancer. A significant association between ACE inhibition and NSCLC (OR, 0.922; 95% CI, 0.816–1.028; *p* = 0.132) was not observed, including lung adenocarcinoma (OR, 1.008; 95% CI, 0.830–1.186; *p* = 0.929) and lung squamous cell carcinoma (OR, 0.917; 95% CI, 0.697–1.137; *p* = 0.440) (Supplementary Table S6). We observed a weak association between ACE inhibition and SCLC (trait: finn-b-C3_SCLC); however, this association was not statistically significant (OR, 1.336; 95% CI, 1.022–1.650; *p* = 0.071). However, after excluding rs118121655 (OR, 1.399; 95% CI, 1.065–1.733; *p* = 0.049) or rs80311894 (OR, 1.411; 95% CI, 1.075–1.747; *p* = 0.045) from the leave-one-out analysis, we found that the association between ACE inhibition and SCLC was statistically significant (Supplementary Table S7).

### 3.3 MR analysis of blood pressure and the risk of lung cancer

To rule out the possibility that ACE inhibition indirectly affects the incidence of lung cancer by reducing SBP, we conducted an MR analysis to explore the relationship between SBP and the risk of lung cancer. Our analysis of the European population failed to demonstrate a statistically significant association between genetically estimated SBP (trait: ieu-b-38) and the incidence of lung cancer (trait: finn-b-C3_BRONCHUS_LUNG) (OR, 0.992; 95% CI, 0.979–1.005; *p* = 0.238; SNPs = 437). Similarly, 38 independently associated SNPs with a genome-wide significance level among Asians (trait: ieu-b-5075; GWAS association: *p* < 5 × 10^−6^) were used as genetic proxies for SBP. Our examination of the Asian population also yielded no significant correlation between genetically estimated SBP and the risk of lung cancer (trait: bbj-a-133) (OR, 0.626; 95% CI, 0.217–1.035; *p* = 0.025; SNPs = 38).

### 3.4 MR analysis of ACEI-induced cough and the risk of lung cancer

**FIGURE 2:**
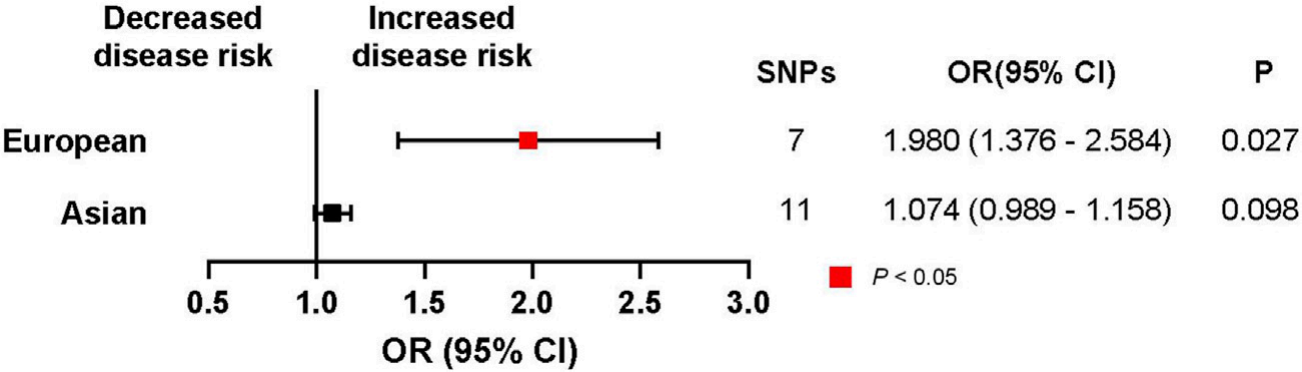
Association of ACEI-induced-cough-related gene expression with lung cancer risk according to the region. CI, confidence interval; P, *p*-value.

**FIGURE 3:**
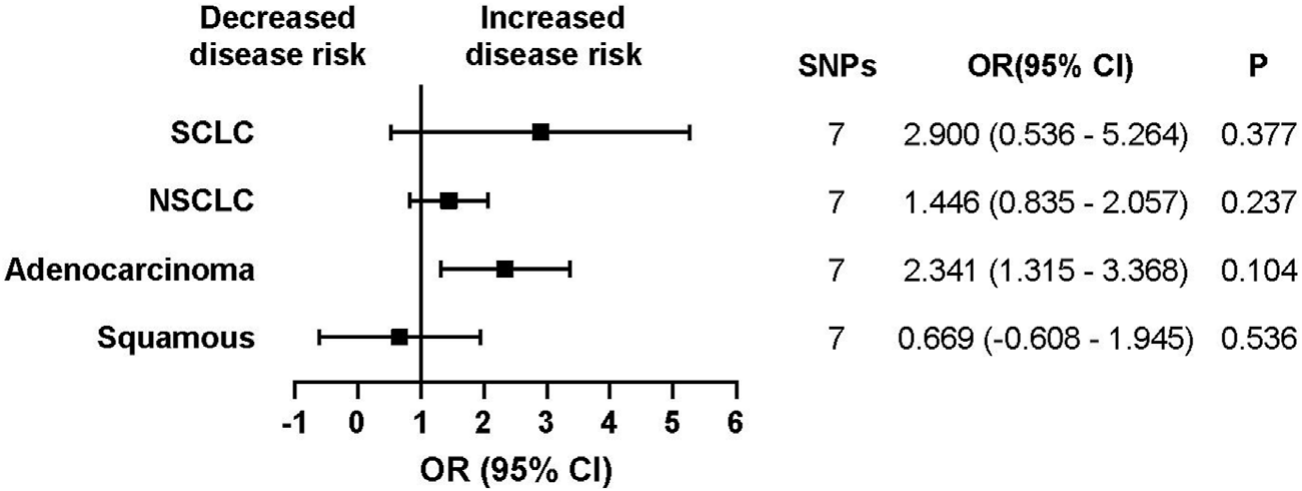
Association between ACEI-induced-cough-related gene expression and different types of lung cancer risk in Europeans. CI, confidence interval; P, *p*-value.

**FIGURE 4:**
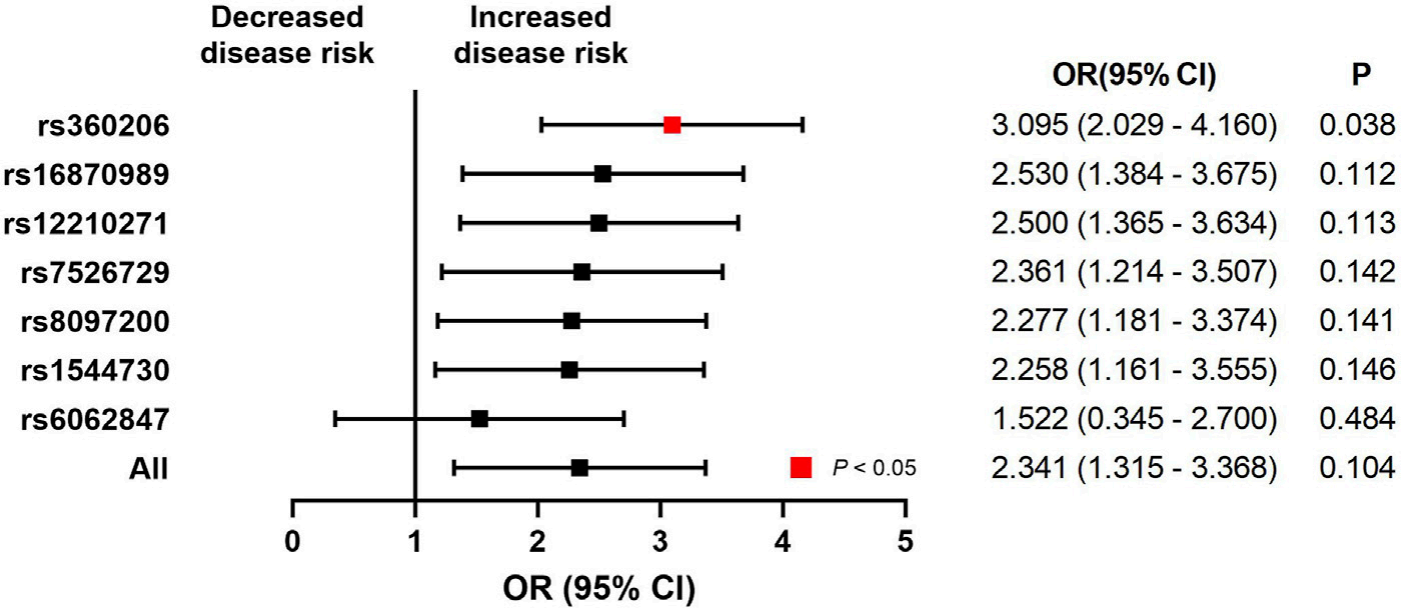
Leave-one-out analysis of the association between ACEI-induced cough and lung adenocarcinoma. CI, confidence interval; P, *p*-value.

An initial analysis of the European cohort was performed (Figure 2). The MR analysis revealed a correlation between ACEI-induced cough and lung cancer (trait: finn-b-C3_BRONCHUS_LUNG) (OR, 1.980; 95% CI, 1.376–2.584; *p* = 0.0266; SNPs = 7). The subgroup analysis of lung cancer pathological subtypes showed no statistically significant association between ACEI-induced cough and the risk of lung squamous cell carcinoma (trait: finn-b-C3_NSCLC_SQUAM) (OR, 0.669; 95% CI, −0.608–1.945; *p* = 0.536; SNPs = 7) (Figure 3). Furthermore, a weak association was observed between ACEI-induced cough and adenocarcinoma (not statistically significant; OR, 2.341; 95% CI, 1.315–3.368; *p* = 0.104; SNPs = 7) (Figure 3). To validate the results, a leave-one-out analysis in which an SNP was excluded from each iteration was performed. When the genetic variant rs360206 was excluded, the formerly weak association between ACEI-induced cough and lung adenocarcinoma was statistically significant (OR, 2.341; 95% CI, 1.315–3.368; *p* = 0.038; SNPs = 6) (Figure 4). Further analyses did not provide evidence suggesting a causal relationship between ACEI-induced cough and other pathological subtypes of lung cancer (Supplementary Table S8).

This analysis was also performed for the Asian cohort (Figure 2), and the results revealed a potential causal relationship between ACEI-induced cough and lung cancer (trait: bbj-a-133) (OR, 1.074; 95% CI, 0.989–1.158; *p* = 0.098; SNPs = 11). The results of the leave-one-out analysis also indicated no causal relationship between ACEI-induced cough and the overall lung cancer incidence among the Asian population. Therefore, an MR analysis of the region subgroup was performed (Figure 2).

### 3.5 Mediation analysis of the association between ACEI-induced cough and lung cancer

**FIGURE 5:**
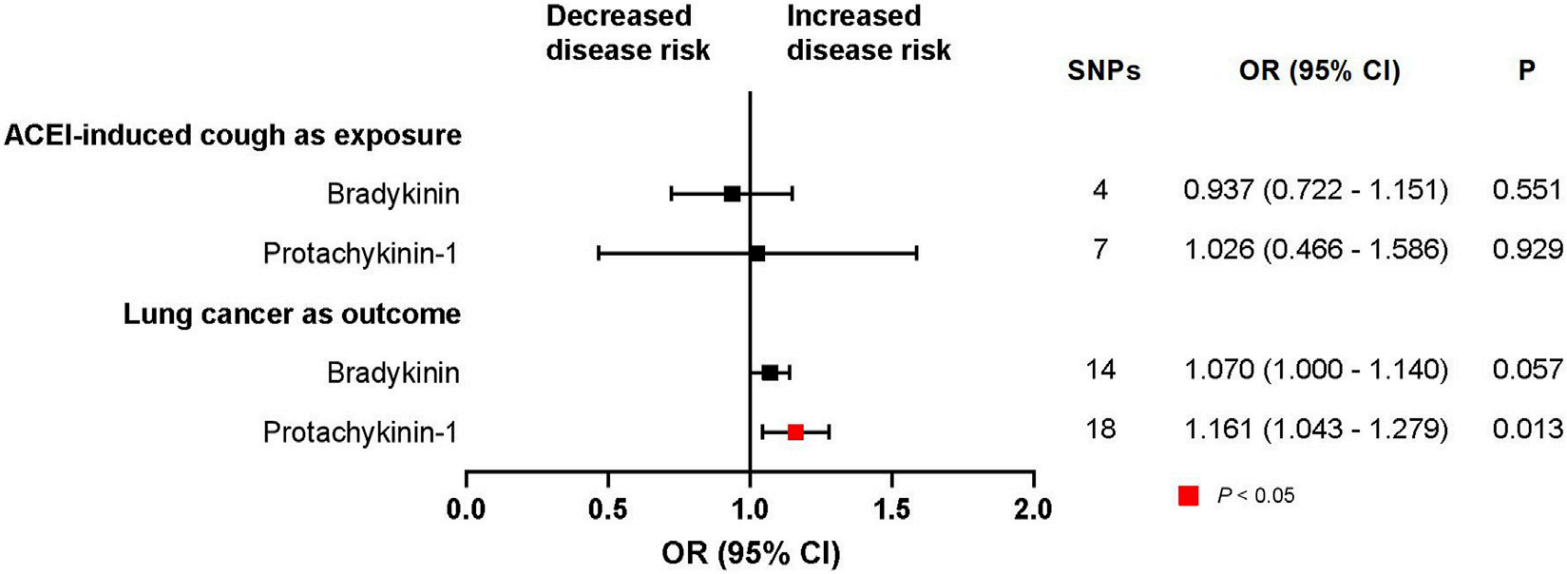
Exploring possible mediators between ACEI-induced cough and lung cancer. CI, confidence interval; P, *p*-value.

We did not identify a statistically significant association between ACEI-induced cough and bradykinin (trait: met-a-656) (OR, 0.937; 95% CI, 0.722–1.151; *p* = 0.551; SNPs = 4). Our findings also failed to demonstrate a statistically significant relationship between bradykinin and the risk of lung cancer (trait: finn-b-C3_BRONCHUS_LUNG) (OR, 1.070; 95% CI, 1.000–1.140; *p* = 0.057; SNPs = 16) (Figure 5). However, a statistically significant association between protachykinin-1 (trait: prot-a-2920) and the risk of lung cancer was established (OR, 1.161; 95% CI, 1.043–1.279; *p* = 0.013; SNPs = 18) (Figure 5). Subsequently, an MR analysis was performed to assess the relationship between ACEI-induced cough and protachykinin-1 (trait: prot-a-2920); however, the results were not statistically significant (OR, 1.026; 95% CI, 0.466–1.586; *p* = 0.929; SNPs = 7) (Figure 5).

In addition, our findings failed to demonstrate a statistically significant relationship between bradykinin and the risk of lung squamous cell carcinoma (trait: finn-b-C3_NSCLC_SQUAM) (OR, 0.838; 95% CI, 0.588–1.087; *p* = 0.527; SNPs = 14) (Supplementary Figure S2). However, a statistically significant association between protachykinin-1 (trait: prot-a-2920) and the risk of lung squamous cell carcinoma was established (OR, 1.298; 95% CI, 1.049–1.548; *p* = 0.040; SNPs = 18) (Supplementary Figure S2). Furthermore, our findings failed to demonstrate a statistically significant relationship between bradykinin and the risk of lung adenocarcinoma (trait: finn-b-C3_NSCLC_ADENO) (OR, 1.321; 95% CI, 0.812–1.829; *p* = 0.283; SNPs = 14). Additionally, no significant association between protachykinin-1 (trait: prot-a-2920) and the risk of lung adenocarcinoma was found (OR, 1.171; 95% CI, 0.955–1.388; *p* = 0.152; SNPs = 18). These findings suggest that bradykinin may not have a causal role in ACEI-induced cough leading to lung cancer or lung squamous cell carcinoma, and that protachykinin-1 may have a causal impact.

### 3.6 Sensitivity analysis

To evaluate the potential pleiotropic effects of the genetic instruments used during this study, we conducted an MR-Egger regression analysis. Our MR analysis (Supplementary Table S9) of the relationship between ACEI-induced cough and lung cancer risk did not reveal any evidence of imbalanced pleiotropy in the genetic instruments (intercept *β* = −0.057, *p* = 0.493 for lung cancer; intercept *β* = 0.058, *p* = 0.866 for SCLC; intercept *β* = 0.015, *p* = 0.850 for NSCLC; intercept *β* = −0.214, *p* = 0.161 for lung adenocarcinoma; and intercept *β* = 0.165, *p* = 0.354 for lung squamous cell carcinoma). To test our IVW hypothesis, we considered these SNPs free of pleiotropy. Because all tests of pleiotropy produced statistically nonsignificant results, thus implying the absence of pleiotropic effects of the genetic instruments, we used the estimates obtained with the IVW method as our primary outcome.

## 4 Discussion

Although ACEIs have prominent therapeutic efficacy when used to treat hypertension and heart failure, their potential adverse effects and association with a higher risk of lung cancer have been controversial. Recently, several clinical studies and meta-analyses have proposed that ACEI use may be linked to a higher risk of lung cancer (Hicks et al., 2018; Wu et al., 2023). However, because of the absence of RCTs, the causal relationship between ACEI use and lung cancer occurrence is uncertain. An increasing number of studies have advocated the MR analysis as a suitable substitute for RCTs. During our MR analysis of six lung cancer cohorts, genetic proxies for ACE inhibition were positively associated with a higher risk of lung cancer. However, this positive association was restricted to Europeans. ACE inhibition is correlated with the occurrence of SCLC (excluding rs118121655 or rs80311894), but not with that of NSCLC. Additionally, the incidence of dry cough, which is one of the most prevalent side effects of ACEI use, is potentially correlated with the risk of lung cancer.

Genetic proxies for ACE inhibition are positively associated with a higher risk of lung cancer among the European population. This finding was consistent with that of previous observational studies (Hicks et al., 2018; Kristensen et al., 2021). However, a meta-analysis reported a significantly increased risk of lung cancer among Asian populations after ACEI use (Wu et al., 2023), in contrast to our MR results, which showed such an association among a European population. This discrepancy may be attributed to the fact that researchers are unable to entirely eliminate or correct confounding factors such as smoking, (Krist et al., 2021), air pollution, (Li et al., 2020), and occupational exposure (Ge et al., 2020; McDowell et al., 2021) in observational studies. Researchers often assume that individuals of different races may experience varying types or degrees of adverse effects of drug treatments in clinical drug trials. A recent study (Huang et al., 2023) suggested that the use of ACEIs may be beneficial for East Asian patients with coronavirus disease 2019 in terms of mortality and a shorter hospital stay. However, the underlying reasons for these phenomena remain unclear, and the data only represent different geographical locations; therefore, they may not accurately reflect the diversity of different races. A previous MR study (Yarmolinsky et al., 2022) that used a GWAS database of European populations to explore the relationship between antihypertensive drugs and cancer reported a negative association. The replicability of these results and their generalizability to other Asian populations are of particular interest. MR analyses effectively addressed the interference of confounding factors at the genetic level. Nevertheless, because observational studies are conducted in the real world, confounding factors are unavoidable, and physicians should vigilantly monitor Asian populations using ACEIs for the possibility of lung cancer.

The MR analysis can serve as an alternative to RCTs by using SNPs as genetic proxies for exposure and random allocations of individuals (Emdin et al., 2017). However, this MR analysis was subject to the following three assumptions: SNPs and ACE inhibition have a robust correlation; no confounder affects ACE inhibition and lung cancer; and that SNPs affect the development of lung cancer only through ACE inhibition, indicating no pleiotropic imbalance.

To fulfill the first assumption, we referred to published MR studies and GWAS and selected genetic instruments associated with the expression of ACE inhibition-related genes at the whole genome level for both Asian and European populations that could be validated in the corresponding population. Based on strict selection criteria, the association between SNPs and ACE inhibition was reliable. To fulfill the second assumption, we conducted literature retrieval and sensitivity analyses that showed no significant association between ACE inhibition and potential confounders. Regarding the third assumption, if the genetic variation associated with ACE inhibition is related to other risk factors that affect cancer development, then the pleiotropic effect of the SNP should yield a positive result (Skrivankova et al., 2021). However, our calculated pleiotropy result was not significant, indicating no pleiotropic effect on the MR analysis that interfered with causal inference. Therefore, our MR analysis almost fulfilled these assumptions, thereby eliminating the influence of confounders and avoiding reverse causality. Compared with the results of a meta-analysis of observational studies, the MR results seem to establish a more reasonable causal relationship. Our findings suggest that ACEI use among the Asian population is not significantly associated with the risk of lung cancer but is positively associated with this risk among the European population (only with SCLC).

Notably, ACE inhibition was associated with the occurrence of SCLC, except for genes rs118121655 or rs80311894; however, the underlying reasons for this association remain unclear. In clinical practice, the detection of mutations in the as rs11655956, rs118138685, rs12150648, rs12452187, rs13342595, rs28656895, rs3730025, rs4365, rs4968771, and rs79480822 genes in patients indicates an increased risk of SCLC after the use of ACEIs. This may provide guidance for the precise use of these drugs.

Although the exact mechanism by which ACE inhibition contributes to the occurrence of lung cancer remains unclear, the genetic proxy for ACE inhibition does not affect the development of lung cancer by reducing SBP. One possible explanation is that ACE inhibition induces the accumulation of bradykinin (Wang et al., 2020) and substance P, (Liau et al., 2019), which have been identified as potential inducers of tumor proliferation (Muñoz and Coveñas, 2014). Consequently, the levels of Ac-SDKP increase (Macconi et al., 2012). Ac-SDKP is an endogenous antifibrotic peptide that has been demonstrated to reduce angiogenesis (Kumar and Yin, 2018).

Because ACEI-induced cough and lung cancer are the two most prominent side effects of ACEIs in the respiratory system, we conducted an exploratory analysis to investigate whether they have a causal relationship. Our study revealed a significant positive correlation between ACEI-induced cough and the risk of lung cancer among a European population. ACEI-induced cough was the result of the suppression of ACE activity, and bradykinin (a degradation product of ACE) accumulated in the lung tissue. However, our mediation MR analysis indicated no discernible relationship between ACEI-induced cough and bradykinin, and no relationship between bradykinin and lung cancer. Further analyses revealed an association between protachykinin-1 (the precursor of tachykinin) and the risk of lung cancer. Tachykinins belong to a family of neuropeptides, including substance P, neurokinin A, neurokinin B, neurokinin K, and neurokinin Y. A previous study indicated that substance P could potentially act as a mediator in the association between ACE inhibition and lung cancer (Muñoz and Coveñas, 2014). Other studies (Fox et al., 1996; Moreaux et al., 2001; Sanchis-Gomar et al., 2020) indicated that ACEIs may induce cough through the ACE-bradykinin-tachykinin or ACE-bradykinin-inflammation pathways, which is an alternative mechanism resulting from the inhibitory effect of ACE inhibition on tachykinin metabolism. However, the mechanism was unclear and requires further molecular biology research.

The MR subgroup analysis of the region revealed no association between ACEI-induced cough and lung cancer risk in the Asian population. During the MR subgroup analysis of the lung cancer pathological subtypes, the association between ACEI-induced cough and lung adenocarcinoma was statistically significant (excluding rs360206). In clinical practice, mutations in genes rs1544730, rs16870989, rs360206, rs6062847, rs7526729, and rs8097200 in patients with ACEI-induced cough indicate a higher risk of lung adenocarcinoma. This finding underscores the current need for individualized and precise treatment.

Although MR is used to address the limitations of observational studies and replace RCTs, this study had certain limitations. First, compensatory processes or feedback mechanisms may dilute genetic effects (Lawlor et al., 2008). Although such compensation may attenuate genetic effects, it could not explain the association between specific genetic proxies of ACE inhibition (or ACEI-induced cough) and the risk of lung cancer. Second, our study incorporated two previously published GWAS datasets of ACEI-induced cough and lung cancer in Asian populations [one from China (Mu et al., 2020) and the other from Japan (Ishigaki et al., 2020)]. Although the heterogeneity of test results was not significant, geographical differences existed between the two populations, and the reliability of the results did not represent all of Asia. Additionally, a previous study (Wu et al., 2023) showed that the duration of medication affects the results of the assessment of the risk of lung cancer for patients using ACEIs. The MR analysis assesses lifelong effects; therefore, the effect size may not be comparable to the short-term effects of using ACEIs. Nevertheless, one of the key strengths of our study was the utilization of existing data obtained from large-scale studies of genetic variations to investigate the effects of ACE inhibition and ACEI-induced cough. This approach circumvents the time and resource limitations associated with RCTs, overcomes potential confounders, and reverses causality limitations associated with standard observational methods, thus lending support to the current clinical guidelines for the treatment of hypertension. Therefore, this MR study is more relevant for assessing the direction of the association than for providing the magnitude of the association, and it is valuable for estimating the long-term effects of ACEI exposure.

In summary, our MR analysis provides robust evidence linking ACEI-induced cough with an increased risk of lung cancer (especially lung adenocarcinoma) in the European population and highlights the necessity for further research to elucidate the underlying mechanisms. Because of the pervasive use of ACEIs, SNP monitoring is a valuable strategy in clinical practice, especially in the presence of side effects. When monitored SNPs are mutated, alternative drugs should be considered instead of ACEIs to avoid the development of lung cancer. Our findings provide further evidence supporting the guidelines that suggest that the occurrence of ACEI-induced cough should be considered an indication for the replacement of ACEIs with angiotensin receptor blockers (Whelton et al., 2018). Our findings have practical implications for minimizing the drug-induced lung cancer risk for patients using ACEIs. Clinicians should consider various options when prescribing drugs to provide the most appropriate choice for their patients. These important findings provide high-quality evidence regarding the long-term safety and risk of adverse events for patients with chronic diseases who use ACEIs.

Our MR analysis found a correlation between ACE inhibition (or ACEI-induced cough) and a higher risk of lung cancer among the European population. Additionally, a correlation was identified between ACEI-induced cough and a higher risk of lung adenocarcinoma. Dry cough has a significant impact on patient compliance with ACEI treatment in the real world; however, high-quality observational studies and RCTs exploring the impact of continued ACEI use on the lung cancer incidence after the development of cough are lacking. Notably, our results provide valuable insights regarding precision medicine for the treatment and management of patients with hypertension. If patients with hypertension experience dry cough as a side effect of ACEI use, then discontinuation of ACEIs and switching to an alternative antihypertensive treatment is recommended.

## Data Availability

The original contributions presented in the study are included in the article/Supplementary Material, further inquiries can be directed to the corresponding author.
