# Supplementary material for: Association between angiotensin-converting enzyme inhibitor-induced cough and the risk of lung cancer: a Mendelian randomization study
Source: Front Pharmacol. 2023 Sep 20;14:1267924. doi: 10.3389/fphar.2023.1267924 (PMC10550256; doi:10.3389/fphar.2023.1267924)
Supplement: Supplementary file 1 [file DataSheet1.DOCX]

**Supplementary Online Content**

**Figure S1.** Possible Explanations for Observed Association in MR Analysis Between ACE Inhibition (or ACEI-induced Cough) and Lung Cancer Risk

**Figure S2.** Exploring possible mediators between ACEI-induced cough and lung squamous cell carcinoma

**Table S1.** Genome-wide association studies used in Mendelian randomisation analysis

**Table S2.** MR association between ACE expression in blood and systolic blood pressure

**Table S3.** Genetic proxies for ACEI-induced cough in Asians

**Table S4.** Genetic proxies for ACEI-induced cough in Europeans

**Table S5.** Leave-one-out analysis of the association between ACE inhibition and lung cancer

**Table S6.** MR analysis of the association between ACE inhibition and specific subtypes of lung cancer

**Table S7.** Leave-one-out analysis of the association between ACE inhibition and small cell lung cancer

**Table S8**. MR analysis of the association between ACEI-induced cough and specific subtypes of lung cancer

**Table S9.** Sensitivity analysis of the association between ACEI-induced cough and lung cancer

**Figure S1.** Possible Explanations for Observed Association in MR Analysis Between ACE Inhibition (or ACEI-induced Cough) and Lung Cancer Risk


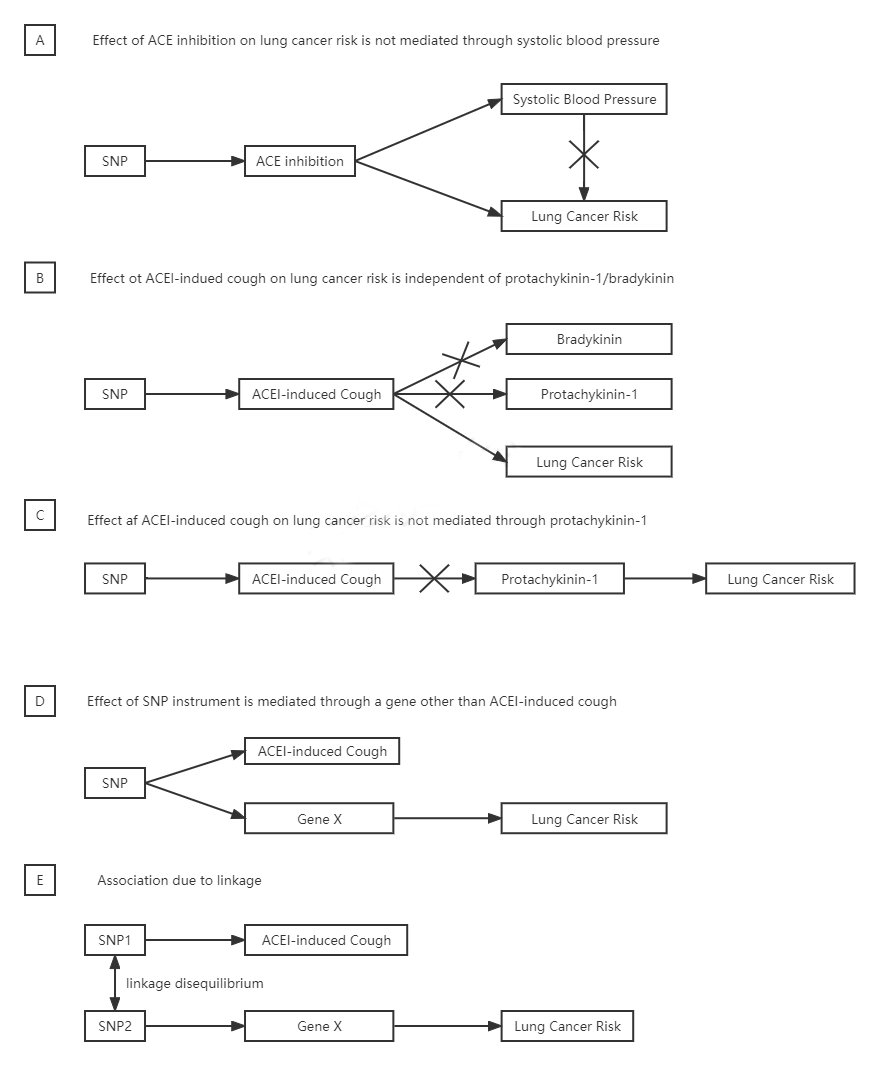


**Figure S2**.Exploring possible mediators between ACEI-induced cough and lung squamous cell carcinoma

**Table S1.** Genome-wide association studies used in Mendelian randomisation analysis

| **Trait** | **Category** | **Disease** | **Number of cases** | **Number of controls** | **Ancestry** | **PMID** | **Authors** | **Publication date** |
| --- | --- | --- | --- | --- | --- | --- | --- | --- |
| ieu-b-38 | Continuous | systolic blood pressure | NA | NA | European | 30224653 | Evangelou, E | 2018 |
| finn-b-C3_BRONCHUS_LUNG | Binary | Malignant neoplasm of bronchus and lung | 1,681 | 217,111 | European | NA | NA | 2021 |
| bbj-a-133 | Disease | Lung cancer | 4,050 | 208,403 | East Asian | 32514122 | Ishigaki K | 2020 |
| finn-b-C3_SCLC | Binary | Small cell lung cancer | 179 | 218,613 | European | NA | NA | 2021 |
| finn-b-C3_LUNG_NONSMALL | Binary | Non-small cell lung cancer | 1,627 | 217,165 | European | NA | NA | 2021 |
| finn-b-C3_NSCLC_ADENO | Binary | Non-small cell lung cancer, adenocarcinoma | 571 | 218,221 | European | NA | NA | 2021 |
| finn-b-C3_NSCLC_SQUAM | Binary | Non-small cell lung cancer, squamous | 365 | 218,427 | European | NA | NA | 2021 |
| [prot-a-2920](https://gwas.mrcieu.ac.uk/datasets/prot-a-2920/) | Immune system | Protachykinin-1 | NA | NA | European | 29875488 | Sun BB | 2018 |
| met-a-656 | Metabolites | Bradykinin, des-arg(9) | NA | NA | European | 24816252 | Shin | 2014 |

Abbreviation: NA,not access.

**Table S2.** MR association between ACE inhibition and systolic blood pressure

| **Exposure** | **SNP** | **coded allele** | **other allele** | **coded allele frequency** | **beta** | **se** | **p-value** | **F-statistic** | **beta** | **se** | **p-value** | **beta** | **se** | **p-value** |
| --- | --- | --- | --- | --- | --- | --- | --- | --- | --- | --- | --- | --- | --- | --- |
| ACE inhibition | rs4343 | A | G | 0.45 | 0.6237682 | 0.02 | 1.53E-213 | 972.7169183 | -0.1125 | 0.0302 | 0.000197401 | -0.180355459 | 0.048415421 | 0.000195187 |
| ACE inhibition | rs12452187 | A | G | 0.6 | 0.2165646 | 0.02 | 2.53E-27 | 117.2505649 | 0.0346 | 0.0315 | 0.2726 | -0.159767571 | 0.145453135 | 0.27202432 |
| ACE inhibition | rs79480822 | C | T | 0.93 | 0.504301 | 0.05 | 6.37E-24 | 101.7277994 | 0.3231 | 0.075 | 1.65402E-05 | -0.640688795 | 0.148720704 | 1.64737E-05 |
| ACE inhibition | rs3730025 | G | A | 0.01 | 0.8035609 | 0.09 | 4.32E-19 | 79.71729877 | -0.0967 | 0.1241 | 0.436 | -0.120339354 | 0.154437579 | 0.435855835 |
| ACE inhibition | rs11655956 | C | G | 0.08 | 0.3207882 | 0.04 | 1.06E-15 | 64.31566829 | EX | EX | EX | EX | EX | EX |
| ACE inhibition | rs118121655 | G | A | 0.96 | 0.5520769 | 0.07 | 3.1E-15 | 62.20181704 | EX | EX | EX | EX | EX | EX |
| ACE inhibition | rs4365 | G | A | 0.97 | 0.5485148 | 0.08 | 7.06E-12 | 47.01070091 | 0.2534 | 0.0857 | 0.00310399 | -0.461974773 | 0.156240087 | 0.003108233 |
| ACE inhibition | rs4968771 | G | A | 0.08 | 0.2016905 | 0.03 | 1.78E-11 | 45.1989531 | EX | EX | EX | EX | EX | EX |
| ACE inhibition | rs12150648 | G | A | 0.96 | 0.3822502 | 0.06 | 1.88E-10 | 40.58755983 | EX | EX | EX | EX | EX | EX |
| ACE inhibition | rs80311894 | T | G | 0.97 | 0.4424648 | 0.07 | 2.6E-10 | 39.95410189 | -0.0827 | 0.0766 | 0.2799 | 0.186907523 | 0.173121116 | 0.280304988 |
| ACE inhibition | rs118138685 | C | G | 0.04 | 0.4175797 | 0.07 | 2.44E-09 | 35.58628691 | -0.1593 | 0.072 | 0.02688 | -0.381484062 | 0.172422175 | 0.026932136 |
| ACE inhibition | rs13342595 | C | T | 0.23 | 0.1192554 | 0.02 | 2.48E-09 | 35.55462607 | -0.1926 | 0.0358 | 7.12607E-08 | -1.615021207 | 0.30019605 | 7.45321E-08 |
| ACE inhibition | rs28656895 | T | C | 0.23 | 0.1178796 | 0.02 | 3.77E-09 | 34.73900024 | -0.1938 | 0.0357 | 5.53898E-08 | -1.64405037 | 0.302851384 | 5.68069E-08 |
| ACE inhibition | rs4968780 | C | A | 0.05 | 0.2812271 | 0.05 | 1.86E-08 | 31.63547271 | -0.1442 | 0.082 | 0.0785995 | -0.512752861 | 0.291579297 | 0.078656252 |

Abbreviation: ACE, Angiotensin-converting enzyme; EX,excluded in the further analysis.

**Table S3.** Genetic proxies for ACEI-induced cough in Asians

| **Trait** | **SNP** | **coded allele** | **other allele** | **coded allele frequency** | **beta** | **se** | **p-value** | **F-statistic** |
| --- | --- | --- | --- | --- | --- | --- | --- | --- |
| ACEI-induced cough | rs6742895 | A | G | 0.0821218 | -0.0751718 | 0.0216689 | 0.000522011 | 12.0 |
| ACEI-induced cough | rs2164796 | A | G | 0.0795437 | -0.0885439 | 0.0219832 | 5.63E-05 | 16.2 |
| ACEI-induced cough | rs34350940 | A | G | 0.0806062 | -0.0925223 | 0.0218491 | 0.000022896 | 17.9 |
| ACEI-induced cough | rs2919051 | T | C | 0.105091 | -0.0688837 | 0.0193989 | 0.000383769 | 12.6 |
| ACEI-induced cough | rs2304663 | T | C | 0.0779492 | -0.0880666 | 0.0221881 | 7.21E-05 | 15.8 |
| ACEI-induced cough | rs9807995 | T | C | 0.0811943 | -0.0911634 | 0.0217771 | 2.83E-05 | 17.5 |
| ACEI-induced cough | rs2304560 | G | A | 0.0798663 | -0.0901775 | 0.0219421 | 3.96E-05 | 16.9 |
| ACEI-induced cough | rs2304561 | A | G | 0.0798277 | -0.090546 | 0.0219468 | 3.69E-05 | 17.0 |
| ACEI-induced cough | rs2118387 | G | A | 0.0795631 | -0.0890039 | 0.0219806 | 5.13E-05 | 16.4 |
| ACEI-induced cough | rs2289192 | C | T | 0.0787635 | -0.0891488 | 0.0220824 | 5.41E-05 | 16.3 |

**Table S4.** Genetic proxies for ACEI-induced cough in Europeans (under LD clumping)

| **Trait** | **SNP** | **coded allele** | **other allele** | **coded allele frequency** | **beta** | **se** | **p-value** | **F-statistic** | **MR_keep exposure (LD clumping)** |
| --- | --- | --- | --- | --- | --- | --- | --- | --- | --- |
| ACEI-induced cough | rs7526729 | A | G | 0.69 | 0.053078443 | 0.009800584 | 0.000000061 | 29.33137612 | TRUE |
| ACEI-induced cough | rs1544730 | A | G | 0.22 | 0.041392685 | 0.005744912 | 5.8E-13 | 51.91351505 | TRUE |
| ACEI-induced cough | rs16870989 | A | T | 0.33 | 0.049218023 | 0.005052257 | 2E-22 | 94.90245806 | TRUE |
| ACEI-induced cough | rs12210271 | T | C | 0.21 | 0.053078443 | 0.005858304 | 1.3E-19 | 82.09043194 | TRUE |
| ACEI-induced cough | rs360206 | C | T | 0.19 | 0.041392685 | 0.006172464 | 2E-11 | 44.97074328 | TRUE |
| ACEI-induced cough | rs8097200 | A | G | 0.83 | 0.049218023 | 0.006343828 | 8.6E-15 | 60.19293651 | TRUE |
| ACEI-induced cough | rs6062847 | T | C | 0.14 | 0.08278537 | 0.006767996 | 2.1E-34 | 149.6190875 | TRUE |

**Table S5.**Leave-one-out analysis of the association between ACE inhibition and lung cancer

| **Exposure** | **GWAS ID(outcome)** | **Outcome** | **SNP** | **beta** | **se** | **p-value** |
| --- | --- | --- | --- | --- | --- | --- |
| ACE inhibition | finn-b-C3_BRONCHUS_LUNG | lung cancer | rs11655956 | -0.044007563 | 0.056253922 | 0.434037029 |
| ACE inhibition | finn-b-C3_BRONCHUS_LUNG | lung cancer | rs118121655 | -0.039716532 | 0.0566458 | 0.483216797 |
| ACE inhibition | finn-b-C3_BRONCHUS_LUNG | lung cancer | rs118138685 | -0.03791525 | 0.054373523 | 0.485608248 |
| ACE inhibition | finn-b-C3_BRONCHUS_LUNG | lung cancer | rs12150648 | -0.043114345 | 0.054189507 | 0.426251976 |
| ACE inhibition | finn-b-C3_BRONCHUS_LUNG | lung cancer | rs12452187 | -0.032038868 | 0.05613204 | 0.568150932 |
| ACE inhibition | finn-b-C3_BRONCHUS_LUNG | lung cancer | rs13342595 | -0.035496185 | 0.05397951 | 0.510804018 |
| ACE inhibition | finn-b-C3_BRONCHUS_LUNG | lung cancer | rs28656895 | -0.035502695 | 0.053963952 | 0.51060464 |
| ACE inhibition | finn-b-C3_BRONCHUS_LUNG | lung cancer | rs3730025 | -0.057820903 | 0.056106287 | 0.302747135 |
| ACE inhibition | finn-b-C3_BRONCHUS_LUNG | lung cancer | rs4365 | -0.058005005 | 0.057367277 | 0.311960637 |
| ACE inhibition | finn-b-C3_BRONCHUS_LUNG | lung cancer | rs4968771 | -0.041544237 | 0.054250734 | 0.443806007 |
| ACE inhibition | finn-b-C3_BRONCHUS_LUNG | lung cancer | rs79480822 | -0.036927662 | 0.058613956 | 0.528684873 |
| ACE inhibition | finn-b-C3_BRONCHUS_LUNG | lung cancer | rs80311894 | -0.045884556 | 0.057046464 | 0.421202636 |
| ACE inhibition | finn-b-C3_BRONCHUS_LUNG | lung cancer | All | -0.042193135 | 0.053313524 | 0.42870173 |

**Table S6.** MR analysis of the association between ACE inhibition and specific subtypes of lung cancer

| **exposure** | **GWAS ID(outcome)** | **Outcome** | **nsnps** | **bIVW** | **seIVW** | **PIVW** | **OR** | **OR(lower)** | **OR(upper)** |
| --- | --- | --- | --- | --- | --- | --- | --- | --- | --- |
| ACE inhibition | [finn-b-C3_SCLC](https://gwas.mrcieu.ac.uk/datasets/finn-b-C3_SCLC/) | Small cell lung cancer | 12 | 0.289506109 | 0.160333 | 0.07097222 | 1.335767601 | 1.021514921 | 1.650020281 |
| ACE inhibition | [ieu-b-4954](https://gwas.mrcieu.ac.uk/datasets/ieu-b-4954/) | Lung cancer | 12 | 9.68E-05 | 0.000273009 | 0.7229036 | 1.000096809 | 0.99956171 | 1.000631907 |
| ACE inhibition | [finn-b-C3_NSCLC_SQUAM](https://gwas.mrcieu.ac.uk/datasets/finn-b-C3_NSCLC_SQUAM/) | Non-small cell lung cancer, squamous | 12 | -0.08676449 | 0.1123452 | 0.4399352 | 0.916893008 | 0.696696416 | 1.1370896 |
| ACE inhibition | finn-b-C3_NSCLC_ADENO | Non-small cell lung cancer, adenocarcinoma | 12 | 0.008044697 | 0.0906783 | 0.9293069 | 1.008077143 | 0.830347675 | 1.185806611 |
| ACE inhibition | [finn-b-C3_BRONCHUS_LUNG](https://gwas.mrcieu.ac.uk/datasets/finn-b-C3_BRONCHUS_LUNG/) | Malignant neoplasm of bronchus and lung | 12 | -0.04219313 | 0.05331352 | 0.4287017 | 0.958684612 | 0.854190113 | 1.063179111 |
| ACE inhibition | finn-b-C3_LUNG_NONSMALL | Non-small cell lung cancer | 12 | -0.081112037 | 0.05391089 | 0.1324379 | 0.922090377 | 0.816425033 | 1.027755722 |

**Table S7.** Leave-one-out analysis of the association between ACE inhibition and small cell lung cancer

| **exposure** | **GWAS ID(outcome)** | **Outcome** | **SNP** | **beta** | **se** | **p-value** |
| --- | --- | --- | --- | --- | --- | --- |
| ACE inhibition | finn-b-C3_SCLC | Small cell lung cancer | rs11655956 | 0.305818697 | 0.169175184 | 0.070652544 |
| ACE inhibition | finn-b-C3_SCLC | Small cell lung cancer | rs118121655 | 0.335646886 | 0.170357803 | 0.048810112 |
| ACE inhibition | finn-b-C3_SCLC | Small cell lung cancer | rs118138685 | 0.285568621 | 0.163515674 | 0.080736323 |
| ACE inhibition | finn-b-C3_SCLC | Small cell lung cancer | rs12150648 | 0.301059357 | 0.162927432 | 0.064629462 |
| ACE inhibition | finn-b-C3_SCLC | Small cell lung cancer | rs12452187 | 0.283067884 | 0.168837696 | 0.093626982 |
| ACE inhibition | finn-b-C3_SCLC | Small cell lung cancer | rs13342595 | 0.264130812 | 0.16233582 | 0.103723448 |
| ACE inhibition | finn-b-C3_SCLC | Small cell lung cancer | rs28656895 | 0.269394724 | 0.162289031 | 0.096920734 |
| ACE inhibition | finn-b-C3_SCLC | Small cell lung cancer | rs3730025 | 0.249677648 | 0.168471159 | 0.138334941 |
| ACE inhibition | finn-b-C3_SCLC | Small cell lung cancer | rs4365 | 0.234200352 | 0.172683832 | 0.175023498 |
| ACE inhibition | finn-b-C3_SCLC | Small cell lung cancer | rs4968771 | 0.27685148 | 0.163145826 | 0.089704757 |
| ACE inhibition | finn-b-C3_SCLC | Small cell lung cancer | rs79480822 | 0.332264404 | 0.17636376 | 0.059568749 |
| ACE inhibition | finn-b-C3_SCLC | Small cell lung cancer | rs80311894 | 0.344385881 | 0.171620412 | 0.044784596 |
| ACE inhibition | finn-b-C3_SCLC | Small cell lung cancer | All | 0.289506109 | 0.160332998 | 0.070972216 |

**Table S8.** MR analysis of the association between ACEI-induced cough and specific subtypes of lung cancer

| **exposure** | **GWAS ID(outcome)** | **Outcome** | **nsnps** | **bIVW** | **se(IVW)** | **PIVW** | **OR** | **OR(lower)** | **OR(upper)** |
| --- | --- | --- | --- | --- | --- | --- | --- | --- | --- |
| ACEI-induced cough | finn-b-C3_NSCLC_SQUAM | Non-small cell lung cancer, squamous | 7 | -0.4024793 | 0.6511057 | 0.5364782 | 0.66866018 | -0.607506992 | 1.944827352 |
| ACEI-induced cough | finn-b-C3_NSCLC_ADENO | Non-small cell lung cancer, adenocarcinoma | 7 | 0.8506276 | 0.5236985 | 0.1043183 | 2.341115675 | 1.314666615 | 3.367564735 |
| ACEI-induced cough | finn-b-C3_SCLC | Small cell lung cancer | 7 | 1.064618958 | 1.206079 | 0.3773927 | 2.899733853 | 0.535819013 | 5.263648693 |
| ACEI-induced cough | finn-b-C3_LUNG_NONSMALL | Non-small cell lung cancer | 7 | 0.3687083 | 0.3118535 | 0.2370817 | 1.445865783 | 0.834632923 | 2.057098643 |

**Table S9.** Sensitivity analysis of the association between ACEI-induced cough and lung cancer

| **exposure** | **GWAS ID(outcome)** | **Outcome** | **nsnps** | **Egger intercept** | P-intercept | P-het IVW | P-het Egger |
| --- | --- | --- | --- | --- | --- | --- | --- |
| ACEI-induced cough | finn-b-C3_NSCLC_SQUAM | Non-small cell lung cancer, squamous | 7 | 0.1650594 | 0.354441 | 0.9136106 | 0.9605754 |
| ACEI-induced cough | finn-b-C3_NSCLC_ADENO | Non-small cell lung cancer, adenocarcinoma | 7 | -0.2144037 | 0.1605269 | 0.5164332 | 0.7758723 |
| ACEI-induced cough | finn-b-C3_SCLC | Small cell lung cancer | 7 | 0.05802816 | 0.866309 | 0.11836817 | 0.07274376 |
| ACEI-induced cough | finn-b-C3_BRONCHUS_LUNG | Malignant neoplasm of bronchus and lung | 7 | -0.05656318 | 0.4929669 | 0.8151843 | 0.7910617 |
| ACEI-induced cough | finn-b-C3_LUNG_NONSMALL | Non-small cell lung cancer | 7 | 0.01539422 | 0.850457 | 0.9396646 | 0.885141 |
